# Optical Coherence Tomography Angiography Imaging in Inherited Retinal Diseases

**DOI:** 10.3390/jcm8122078

**Published:** 2019-11-28

**Authors:** Sally S. Ong, Tapan P. Patel, Mandeep S. Singh

**Affiliations:** Wilmer Eye Institute, Johns Hopkins Hospital, Baltimore, MD 21287, USA; ssallyong@gmail.com (S.S.O.); tpatel29@jhmi.edu (T.P.P.)

**Keywords:** optical coherence tomography angiography, degenerative retinal diseases, retinitis pigmentosa, Stargardt disease, Best disease, choroideremia, gene therapy, retinal pigment epithelium, in vivo imaging, choriocapillaris

## Abstract

Optical coherence tomography angiography (OCTA) is a novel, noninvasive imaging modality that allows depth-resolved imaging of the microvasculature in the retina and the choroid. It is a powerful research tool to study the pathobiology of retinal diseases, including inherited retinal dystrophies. In this review, we provide an overview of the evolution of OCTA technology, compare the specifications of various OCTA devices, and summarize key findings from published OCTA studies in inherited retinal dystrophies including retinitis pigmentosa, Stargardt disease, Best vitelliform macular dystrophy, and choroideremia. OCTA imaging has provided new data on characteristics of these conditions and has contributed to a deeper understanding of inherited retinal disease.

## 1. Introduction

Optical coherence tomography angiography (OCTA) is a non-invasive imaging modality that utilizes motion contrast imaging of blood flow to generate volumetric cross-sectional angiographic images of the retina [1,2,3]. It has the potential to image the retinal and choroidal vascular networks with an unpreceded level of detail. With this retinal and choroidal vascular imaging capability, OCTA could significantly enhance our understanding of retinal diseases. 

OCTA compares the decorrelation signal between sequential OCT b-scans taken at exactly the same cross-section, in order to separate moving scatters from static background tissue, to generate an angiogram. Essentially, it is a motion-contrast image. *En* face OCT angiograms are co-registered with corresponding OCT b-scans. This enables blood flow and structural information to be viewed at the same time. OCTA can be used to visualize and localize abnormal microvasculature in diseases that affect the central macula, such as age-related macular degeneration, diabetic maculopathy, retinal vascular occlusion, and macular telangiectasia [4,5,6,7,8]. Although inherited retinal diseases (IRDs) are not primarily vascular diseases, OCTA is being used to study their vascular-related phenotypic aspects of IRD. In this review, we summarize the evolution of OCTA technology, compare the specifications of currently available OCTA devices, and provide a concise overview of the OCTA findings in IRD, including retinitis pigmentosa, Stargardt disease, Best vitelliform macular dystrophy and choroideremia. 

## 2. Comparison of OCTA and Conventional Angiography

The ability to visualize and quantify retinal and choroidal vascular networks is important in day-to-day clinical decision making, and in research endeavors to better understand the pathophysiology of disease. The systemic administration of fluorescein and indocyanine green (ICG) dyes are widely used in conventional (dye-based) angiography to visualize the retinal and choroidal vasculature, respectively. With the advent of ultra-wide-field dye angiography, peripheral capillary nonperfusion, neovascularization, and earlier stages of retinopathy can be readily identified. There are, however, several limitations of conventional angiography. First, fluorescein angiography (FA) and ICG angiography (ICGA) require intravenous dye injection. These dyes can cause adverse side effects that include nausea, vomiting, urticaria and even anaphylaxis. Second, especially in diseases with retinal pigment epithelial atrophy, dye leakage can be difficult to ascertain against the background of window defects. Finally, these tests yield a two-dimensional photograph of the retina with little depth information, so the anatomical depth localization of a choroidal neovascular membrane, (e.g., type I vs. type II), is somewhat limited. 

OCTA has several advantages over conventional dye-based angiography. OCTA is noninvasive, fast, and dye-free, and provides high-resolution depth-resolved and three-dimensional anatomical details of the retinal and choriocapillaris vascular networks. In addition, quantification of vessel density, and blood flow, in the different layers of the retina is possible with OCTA. Relative disadvantages are that most current OCTA systems provide either a 3 mm × 3 mm or 6 mm × 6 mm limited field of view of the retina. The image resolution is diminished with increasing scanning area. Although OCTA provides excellent anatomic details of normal and abnormal vascular networks, functional information about actual leakage (i.e., direct evidence of the integrity of blood–retinal barrier) can only be obtained via conventional angiogram. Furthermore, OCTA typically is more sensitive to blink and motion artifacts than conventional angiogram. 

OCTA technology is relatively new, but has evolved rapidly. The first commercially available OCTA machines in clinical practice only became available in 2016, so there is a need to further understand the potential role of OCTA in the diagnosis and follow up of various retinal diseases, particularly in terms of its role in influencing treatment decisions. Modern-day OCTA devices all rely on Fourier-domain OCT implementation, i.e. spectral-domain and swept-source, which provides significantly higher acquisition speed than time-domain OCT. Barton et al. [9], in 2005, adapted speckle analysis on time-domain OCT to generate an angiogram based on amplitude or intensity. Speckles are a property of the interferometric nature of OCT. The speckle pattern stays relatively constant over time for static objects while the pattern changes for objects in motion. Therefore, analysis of speckle variation contains information regarding the motion of scatterers, i.e. erythrocytes. In 2009, Wang et al. [10] described a novel imaging technique, named optical microangiography (OMAG) in which spatial frequency analysis of time-varying spectral interferograms was used to separate signals that are backscattered by moving particles from signals that are backscattered by static particles to generate a high-resolution angiogram image. Subsequently, Jia et al. [11], in 2012, developed an efficient signal processing algorithm, termed split-spectrum amplitude-decorrelation angiography (SSADA), to decrease the pulsatile bulk motion noise and improve the signal-to-noise ratio of flow detection. In this method, the full OCT spectrum is split into several narrower bands, and inter-B-scan decorrelation computed and then averaged. 

The pathway to clinical usability of OCTA was cleared when Optovue, Inc acquired SSADA intellectual property and incorporated orthogonal registration and tracking on their commercial SD-OCT platform in 2012, and when they obtained FDA approval and U.S. commercial availability in 2016. In parallel, Carl Zeiss Inc. incorporated OMAG technology in their eye-tracking enabled SD-OCT system in 2013 and launched a commercial product in 2015. Several other manufacturers, including Topcon and Heidelberg, developed OCTA prototypes in 2014 and also gained FDA approval for commercial OCTA machines in 2016. 

## 3. OCTA Devices

Currently, there are four FDA-approved OCTA devices for commercial and clinical use in the USA, that are manufactured by Zeiss, Optovue, Topcon, and Heidelberg. At the time of writing, Nidek and Canon OCTA devices are not available in the USA market. A comparison of technical specifications of the six OCTA devices, based on publicly available brochures and manuals, are presented in Table 1. The software for visualization of volumetric data, and segmentation algorithms, appear to differ significantly between devices. Li et al. [12] compared the image quality, vessel visibility, and motion artifacts of 27 patients across four OCTA systems, and found significant variability in clinical performance. In this small sample study, among the devices that were tested, AngioVue had the highest quality of vessel visibility, and the fewest motion artifacts.

## 4. Disease Context of Inherited Retinal Diseases

With the advent of OCTA, it is now possible to study the hemodynamics of individual retinal and choriocapillaris vascular layers noninvasively. This may help improve our understanding of the pathobiology of inherited retinal diseases like retinitis pigmentosa (RP), Stargardt disease (STGD), Best vitelliform macular dystrophy (BVMD), and choroideremia (CHM). Quantification of vascular changes using OCTA may also be used as an objective measure to monitor microvasculature changes during disease progression. In recent years, several published studies have examined vascular perfusion changes in RP, STGD, BVMD, and CHM as compared to healthy age-matched controls.

RP is the most frequently inherited retinal dystrophy, affecting 1 in 4000 people globally [13]. It can be inherited as an autosomal recessive (50–60%), autosomal dominant (30–40%) or X-linked recessive (5–15%) disorder [13]. The earliest sign of the disease includes night blindness. This is followed by progressive mid-peripheral and peripheral loss of vision, and eventually central loss of vision [13]. Clinical manifestations include waxy pallor of the disc, attenuation of retinal vessels and bone spicule intraretinal pigmentation [13]. Although clinical features and visual field findings are helpful, electroretinogram (ERG) is necessary to establish an accurate diagnosis. ERG demonstrates diminished rod and cone response amplitudes and a delay in their latency [13]. RP has classically been defined as primary degeneration of rod photoreceptors followed by secondary degeneration of cone photoreceptors [14]. However, histopathologic studies have demonstrated that in addition to photoreceptor loss, the choriocapillaris and retinal pigment epithelium (RPE) are also affected in RP [15].

In contrast, STGD is the most common form of inherited macular dystrophy and affects 1 in 10,000 people worldwide [16]. STGD can be associated with many different genetic mutations but is most commonly associated with mutations in the *ABCA4* gene on chromosome 1, which is inherited in an autosomal recessive manner [17]. STGD causes a progressive bilateral loss of central vision and its onset can be variable: early onset at age 10 or younger is typically associated with more severe disease and a rapid decline in visual acuity [18], while late onset at 45 years or older can be associated with milder disease and preserved visual acuity for many years [19]. Clinical presentation includes a beaten bronze or bulls’ eye macular appearance and characteristic deep yellowish-white pisciform flecks in the macular and perimacular region [20]. Loss of function of proteins encoded by the *ABCA4* gene is thought to cause excessive accumulation of toxic lipofuscin in the lysosomal compartment of RPE cells, which leads to primary RPE atrophy [21]. The RPE and choroid are thought to be a coadjutant functional complex and histopathologic studies have found choriocapillaris loss in areas of RPE atrophy in STGD [22,23,24].

BVMD is an inherited macular dystrophy with a typical onset in childhood, and sometimes later in adolescence. The exact prevalence is unknown [25]. It is most commonly an autosomal dominant disease, caused by a mutation in the *BEST1* gene, which encodes bestrophin-1, a transmembrane protein located on the basolateral aspect of RPE cells. Clinically, patients can present with characteristic bilateral yolk-like lesions in the macula. In patients with BVMD, the electrooculogram is usually abnormal, with an Arden ratio less than 1.5, while the full field ERG is normal [25]. Meanwhile, CHM is an X-linked chorioretinal dystrophy characterized by progressive degeneration of the RPE, photoreceptors and choriocapillaris [26]. The prevalence of CHM has been estimated to range from 1 in 50,000 to 100,000 [27]. The disease is due to mutations in the *CHM* gene located at Xq21.2, which encodes Rab escort protein 1 (REP1) [26]. Disease onset is usually in childhood but some patients maintain good visual acuity for 40–50 years. Clinical findings include mottled pigmentation in the anterior equatorial region and macula in early stages of disease, and confluent scalloped areas of RPE and choriocapillaris loss with preservation of larger choroidal vessels in the later stages of disease [27].

## 5. OCTA in Retinitis Pigmentosa

**Figure 1:**
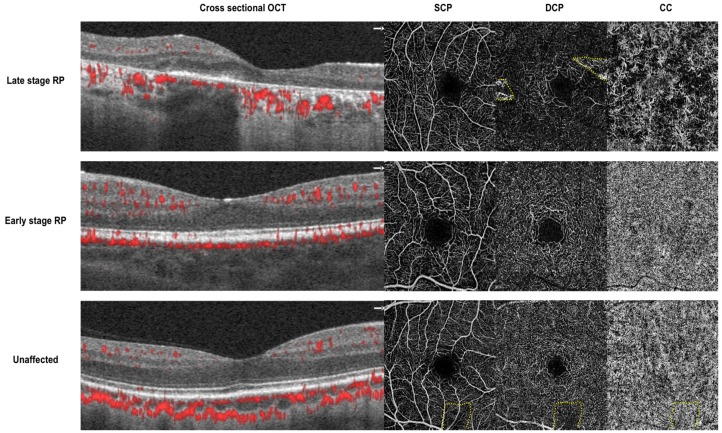
Cross sectional optical coherence tomography (OCT) with angio flow (denoted in red) and 3 × 3mm *en* face optical coherence tomography angiography (OCTA) of the superficial capillary plexus (SCP), deep capillary plexus (DCP) and choriocapillaris (CC). Top—images from a 28-year-old man with severe center involving retinitis pigmentosa (*RP1* mutation). There is diffuse loss of vasculature in the DCP and CC. Middle—images from an 18-year-old woman with mild center sparing retinitis pigmentosa (*IMPDH1* mutation). The vasculature in SCP, DCP, and CC appear grossly preserved. Bottom—images from a 44-year-old male control without retinitis pigmentosa. Dotted yellow lines denote artifacts from segmentation errors (top) and a vitreous floater (bottom).

Multiple groups have found that the retinal and choriocapillaris microvasculature is affected in RP. A summary of studies examining these parameters is provided in Table 2. Examples of OCTA applied to image the retinal and choriocapillaris microvasculature status in patients with RP is shown in Figure 1.

Most studies have shown that vascular flow in the superficial and deep retinal capillary plexuses are lower in RP patients when compared to controls. Toto et al., Battaglia Parodi et al. and Alnawaiseh et al. found that the superficial and deep capillary plexus vessel density in the whole *en* face image were significantly lower in RP patients as compared to controls [28,29,30]. Sugahara et al. similarly found a significantly lower superficial and deep capillary plexus vessel density in the parafoveal region in RP patients vs. controls [31]. Meanwhile, Koyanagi and coauthors reported a lower flow density in the foveal and parafoveal superficial capillary plexuses and parafoveal deep capillary plexus but not in the foveal deep capillary plexus in RP eyes [32]. Inooka and colleagues described decreased perfusion density and vessel length density in the superficial and deep capillary plexus and whole retina in RP eyes compared to controls [33]. Takagi et al. examined flow area in both the superficial and deep capillary plexus and similarly showed that these parameters were lower in RP eyes vs. controls [34]. Wang and colleagues examined the foveal and parafoveal subfields and found that the vessel area densities in the superficial capillary plexus in all subfields in RP eyes were lower than in control eyes [35].

In comparison, Hagag et al. found reductions in vessel density in the perifoveal deep retinal plexus (ICP and DCP), but not in the superficial vascular complex (SVC), in RP eyes [36]. They also demonstrated a significant correlation between outer retinal thickness with intermediate capillary plexus (ICP) as well as DCP vessel density, but not in the SVC, in eyes without cystoid macular edema (CME) [36]. As explained by the authors, one of the possible reasons for the difference between their results and other published studies is that the commercial software used in most other studies faultily places the ICP with the superficial plexus slab. In contrast, Hagag and coauthors used a reflectance based projection resolved OCTA algorithm, that suppressed projection artifacts and divided retinal vasculature into three distinct layers in the macula [36]. Interestingly, an earlier qualitative study by Rezaei and colleagues examining 25 eyes from 13 RP patients with varying degrees of visual field loss reported that eyes with central visual field less than 30 degrees demonstrated abnormal microvasculature in the deep retinal and choriocapillaris layers. The superficial retinal layer was only affected in end stage eyes [37].

Many of the same studies also compared choriocapillaris flow in RP patients vs. controls, and the results are mixed. Toto et al. and Alnawaiseh et al. demonstrated lower choriocapillaris flow density [29,30]. and Guduru showed that the number and area of flow voids in the choriocapillaris were higher in RP eyes when compared to controls [29]. A qualitative study using wide angle swept source OCTA by Miyata and colleagues, with 43 RP eyes and 12 healthy eyes, demonstrated that concentric and vermicular choriocapillaris flow deficits were observed in 23% and 40% of RP eyes, respectively, and 0% of healthy eyes [38]. However, other studies showed no difference in choriocapillaris flow parameters between RP and control eyes [28,31,34]. Some authors have attributed this discrepancy to the size of OCTA images captured. Narrow angle OCTA with dimensions of 3 × 3 mm is thought to miss choriocapillaris changes that occur outside the macular center [38].

In addition, many groups have also examined foveal avascular zone (FAZ) size, and most agree that the FAZ area is larger in RP eyes when compared to controls [29,31,33,35]. Notably, Battaglia Parodi et al. and Takagi et al. examined the FAZ in the superficial and deep capillary plexuses separately, and while Battaglia Parodi and coauthors found that the deep FAZ but not the superficial FAZ is larger in RP eyes [28], Takagi et al. found that the superficial but non-deep FAZ is larger in RP eyes [34]. In contrast, Koyanagi and colleagues found that neither the superficial nor deep FAZ was significantly different between RP and control eyes [32]. 

Significant associations between visual acuity and parafoveal flow density in the superficial [29,32] and deep retinal layer [31,32], foveal flow density in the superficial retinal layer [32,35], and superficial FAZ [31,32,35] have also been reported. Correlations between superficial and deep capillary plexus vessel densities with multifocal electroretinogram values and ganglion cell complex layer thickness have also been shown [30]. One study examined OCTA progression over time and found that perfusion density decreased significantly at the superficial and deep capillary plexus (2.42% ± 0.62% and 2.41% ± 0.76% per year) and FAZ area increased significantly at the superficial and deep layers (0.078 ± 0.021 mm^2^ and 0.152 ± 0.039 mm^2^ per year) (all *p* < 0.005) [39]. 

## 6. OCTA in Stargardt Disease

Retinal and choriocapillaris microvascular abnormalities have also been reported in STGD patients when compared to controls (Table 3). Battaglia Parodi et al. demonstrated decreased vessel densities in the superficial and deep capillary plexuses, as well as in the choriocapillaris, in STGD eyes vs. controls [40]. The authors also found a larger superficial FAZ size in STGD patients than controls [40]. Mastropasqua and coauthors reported decreased vessel densities in the superficial capillary plexus (parafoveal), deep capillary plexus (foveal and parafoveal), and choriocapillaris (foveal and parafoveal), in STGD eyes when compared to controls [41]. Alabduljalil compared 23 STGD patients and 10 normal patients, and found a lower total choriocapillaris vessel density in STGD patients (92.0 ± 0.2) than normal subjects (99.0 ± 0.19, *p* = 0.0044) [42]. The total choriocapillaris vessel density was also found to be correlated with greater areas of total inner segment/outer segment photoreceptor (IS/OS) loss, total retinal pigment epithelium (RPE) loss, matched degeneration, and isolated IS/OS loss, but not with isolated RPE atrophy [42]. These data suggested that choriocapillaris atrophy was significantly associated with photoreceptor and RPE atrophy.

Guduru et al. compared RPE atrophy on fundus autofluorescence imaging and hypointensity at the choriocapillaris layer on OCTA in 22 STGD patients (43 eyes), and found that the RPE atrophy area (6.7 ± 4.4 mm^2^) was larger than the choriocapillaris hypointense area (4.2 ± 3.6 mm^2^) (*p* = 0.004). Those data suggested that RPE damage may precede choriocapillaris atrophy in STGD [43]. Functionally, Mastropasqua and colleagues examined 17 eyes from 9 patients with STGD and found that the percent perfused choriocapillaris area was associated with retinal sensitivity analyzed by microperimetry (*p* < 0.001), thereby suggesting the possibility of utilizing choriocapillaris dysfunction as a predictor for retinal function in STGD patients [44].

## 7. OCTA in Best Vitelliform Macular Dystrophy

OCTA abnormalities in BVMD have been reported. In a study by Guduru et al. [45], 19 eyes from 10 patients with BVMD were imaged using Topcon DRI OCT Triton (swept source 3D OCT, 6 × 6 mm and 3 × 3 mm field of view around the macula). The majority of patients had an abnormal FAZ in the superficial (74%) and deep (100%) retinal layers. Patchy vascularity loss in the superficial and deep layers of retina was also noted, along with hyporeflective center in the choriocapillaris layer that was likely attributable to shadowing from overlying hyperreflective vitelliform material. Patients with CNV had, in addition, hyperreflective material within the hyporeflective center in the choriocapillaris layer. The authors concluded that OCTA is superior to FA in detecting CNV because the vitelliform material masks CNV on FA, whereas OCTA allows examination of vessels across different layers of the retina and choroid. This study did not specify the stage of BVMD so it is unclear whether the OCTA abnormalities reported are generalizable throughout the course of BVMD.

A separate study by Wang et al. [46] reported OCTA findings in 22 eyes of 11 BVMD patients, imaged with the Optovue RTVue Avanti machine (3 × 3mm scans). Patients were grouped into either a vitelliform group (stages 1–4) or a postvitelliform group (stage 5, atrophic/cicatricial). All patients had reduced superficial vascular flow density (whole: 49.2% vs. 53.9%, *p* < 0.001) and choriocapillaris flow area (5.1 vs. 5.5mm^2^, *p* = 0.02) compared to normal subjects. The choriocapillaris in the vitelliform group showed hypointense signal in the choriocapillaris due to signal blockage; in later stages of BVMD (stage 3–4), the hypointense signal was interspersed with hyperintense signal likely due to RPE atrophy. In eyes with stage 5 lesions, medium and large choroidal vessels were visible due to atrophy of the RPE and choriocapillaris. 

Battaglia Parodi and colleagues also compared 66 eyes of 33 patients with BVMD with controls using the Topcon DRI OCT Triton (4.5 × 4.5 mm) [47]. Patients were divided into Gass’ five stages of disease. The authors found that stages 3–4 and 5 eyes had significant reductions of SCP (0.37 ± 0.07; *p* < 0.0001 and 0.37 ± 0.09; *p* = 0.02 respectively) and DCP flow (0.38 ± 0.05; *p* < 0.0001 and 0.38 ± 0.03; *p* = 0.0004 respectively) compared to controls (0.43 ± 0.02 and 0.44 ± 0.02 respectively). They also reported FAZ enlargement at the DCP (*p* = 0.001) in BVMD patients compared to controls and significant correlations between DCP vessel density with stage and best corrected visual acuity. The authors further identified CNV in one third of their BVMD eyes, with stages 4 and 5 eyes being affected the most (88%).

## 8. OCTA in Choroideremia

OCTA imaging of patients with choroideremia has elucidated the changes in choriocapillaris anatomy that were previously unrecognized by conventional angiography or OCT imaging. Jain et al. [48]. reported the OCTA findings in 14 eyes of 7 males with choroideremia, 4 eyes of 2 female carriers and 6 eyes of 6 controls. Images were acquired with the Optovue Avanti RTVue XR device. The mean macular choriocapillaris density was lower in the choroideremia group (82.9% ± 13.4%), compared to carriers (93.0% ± 3.8%) and controls (98.2% ± 1.3%). Interestingly, the choriocapillaris density was higher in regions with preserved EZ in both affected males and carrier females. Furthermore, the most severely affected eyes had a distinct transition zones between preserved and diseased choriocapillaris, whereas carrier eyes had patchy, poorly defined regions of CC loss. 

Battaglia Parodi and coauthors utilized the DRI OCT Triton (3 × 3 and 6 × 6 mm scans) to compare 12 eyes from 6 CHM patients with controls [49]. They reported that there was no difference in SCP vessel density among patients and controls, even when the preserved central island and external area were analyzed separately. However, statistically significant differences were found for DCP and choriocapillaris vessel densities when compared to controls. The overall, preserved central island, and external area DCP vessel densities were attenuated in patients with CHM (0.027 ± 0.002, 0.037 ± 0.02, and 0.017 ± 0.02 respectively; all *p* < 0.01) when compared to controls (0.43 ± 0.026, 0.43 ± 0.03, and 0.43 ± 0.03, respectively). In comparison, the overall, and external area choriocapillaris vessel densities were reduced in patients with CHM (0.147 ± 0.04 and 0.0 ± 0.0 respectively; all *p* < 0.01) when compared to controls (0.496 ± 0.02 and 0.49 ± 0.02 respectively) but there was no difference in the preserved island alone when comparing patients and controls (*p* = 0.64). These results suggest that SCP vasculature is unaffected in CHM, the DCP vasculature is affected in all areas including the central preserved island while the choriocapillaris vasculature is unaffected when the central RPE is still preserved. 

In contrast, Abbouda et al. [26] assessed changes in the superficial retinal vessel network in CHM (Optovue Avanti RTVue XR; 6 × 6 mm scans) and showed a significantly reduced area in CHM compared to carriers and controls (12.93 ± 2.06 mm^2^ in CHM, 15.36 ± 0.60 mm^2^ in carrier subjects, and 15.30 ± 1.35 mm^2^ in controls). A total of 17 eyes from 9 male patients and 9 eyes from 5 female carriers were examined, with 14 normal subjects also enrolled. The mean choriocapillaris area with flow was also reduced (6.97 ± 5.26 mm^2^ in CHM subjects, 21.65 ± 0.17 mm^2^ in carriers and 21.36 ± 0.76 mm^2^ in controls). The choriocapillaris area with flow was positively correlated with the superficial vessel area, however, it is unclear why superficial retinal vascular changes occur in CHM when the central retinal tissue is still anatomically preserved. The authors postulated that a reduction in choriocapillaris flow causes a compensatory reduction in superficial retinal circulation, in order to maintain a balance between the retinal and choroidal circulations.

In later stages of CHM, choroidal neovascularization can develop, which can be difficult to detect on conventional angiogram. Patel et al. demonstrated OCTA evidence of CNV with high flow signal in sub-RPE fibrovascular tissue [50]. Given the rarity of this disease and somewhat contradictory results in some of the above studies, additional OCTA studies enrolling larger number of patients are needed. In particular, clinical trials for treating CHM utilizing retinal gene replacement therapy are currently underway [51] and it will be important to be able to monitor how the choriocapillaris and FAZ areas, along with retinal vessel density recover with treatment. OCTA may be an important quantitative tool for following response to treatment. 

## 9. Conclusions

OCTA is a useful modality to evaluate retinal and choroidal blood flow in patients with IRDs, including RP, STGD, BVMD, and CHM. OCTA imaging has yielded new insights into the occurrence of vascular insufficiency in these conditions. Using OCTA to study retinal and choroidal blood flow in patients with IRDs may reveal further insights into the pathogenesis and natural history of disease in these conditions. The role of OCTA imaging in the clinical management of patients with IRDs is yet to be defined clearly.

## Figures and Tables

**Table 1:** Comparison of optical coherence tomography angiography (OCTA) systems and their specifications.

OCTA System	OCT Model	Optical Source, nm	Scan Speed	Resolution (Axial × Transverse, Microns)	Imaging Depth, mm	Imaging Size, mm	Imaging Volume	Theoretical Acquisition Time, s
Zeiss AngioPlex	Cirrus HD-OCT 5000; SD-OCT	840	68,000	5 × 15	2	3 × 3, 6 × 6	245 × 245 or 350 × 350	3.6
Optovue AngioVue	RTVue XR AVANTI; SD-OCT	840	70,000	5 × 15	2.0–3.0	3 × 3, 6 × 6, 8 × 8	304 × 304 or 400 × 400	3
Topcon Triton	DRI Triton; SS-OCT	1050	100,000	8 × 20	2.6	3 × 3, 6 × 6	256 × 256, 320 × 320	2.7
Heidelberg Spectralis	Spectralis OCT2; SD-OCT	870	85,000	5 × 6	2	3 × 3	256 × 256, 512 × 512	5.4
Nidek AngioScan	RS-3000 Advance; SD-OCT	880	53,000	7 × 20	2.1	3 × 3 to 9 × 9	256 × 256	2.5
Canon Angio eXpert	OCT-HS100; SD-OCT	855	70,000	no data	no data	3 × 3 to 8 × 8	no data	3

**Table 2:** Summary of Microvascular Changes Reported in the Literature Comparing Retinitis Pigmentosa Patients and Unaffected Controls.

OCTA	Size of OCTA Images (mm)	Groups	N, Eyes (n with CME ^+^)	Age		BCVA		SCP VD *		DCP VD *		CC VD		FAZ Size	
				Mean Years ± SD	*p*-Value	Mean logMAR ± SD	*p*-Value	Mean % ± SD	*p*-Value	Mean % ± SD	*p*-Value	Mean % ± SD	*p*-Value	Mean mm^2^ ± SD	*p*-Value
Toto, 2016 (*p*)															
Optovue	3 × 3	RP	26	40.1 ± 7.3	1.0	0.5 ± 0.2	<0.001	42.2 ± 3.4	<0.001	42.7 ± 6.2	<0.001	65.3 ± 2.7	0.02	NA	
		Control	24	42.2 ± 6.5		0.0 ± 0.0		51.4 ± 2.3		56.6 ± 2.2		67.2 ± 1.4			
Battaglia Parodi, 2016 (*p*)															
Triton	3 × 3	RP	32 (2)	53 ± 18	NA	0.5 ± 0.3	NA	29.5 ± 6.8	0.009	28.7 ± 7.5	0.001	51 ± 4.4	0.7	Sup: 0.28 ± 0.13 Deep: 0.54 ± 0.21	Sup: 0.4 Deep: 0.001
		Control	30	53 ± 17		0.0 ± 0.0		34.1 ± 4.3		35.5 ± 5.7		51.3 ± 2.2		Sup: 0.24 ± 0.13 Deep: 0.24 ± 0.16	
Sugahara, 2017 (*p*)															
Optovue	3 × 3	RP	68 (0)	49.9 ± 17.6	0.25	0.16 ± 0.38	<0.001	Parafoveal: 47.0 ± 4.9	<0.001	Parafoveal: 52.4 ± 5.5	<0.001	61.1 ± 2.8	0.3	Sup: 0.34 ± 0.20 Deep: 0.43 ± 0.15	Sup: 0.03 Deep: 0.02
		Control	32	54.4 ± 19.9		−0.11 ± 0.09		Parafoveal: 55.1 ± 3.1		Parafoveal: 60.4 ± 3.1		61.5 ± 1.4		Sup: 0.28 ± 0.08 Deep: 0.356 ± 0.114	
Alnawaiseh, 2017 (*p*)															
Optovue	6 × 6	RP	20 (0)	42.4 ± 14.1	0.917	0.54 ± 0.38	<0.001	43.8 ± 4.6	<0.001	43.4 ± 6.7	<0.001	85.6 ± 11.9	0.005	0.5 ± 0.29	0.02
		Control	21	41.5 ± 13.5		0.0 ± 0.06		52.8 ± 2.9		60.0 ± 3.0		94.4 ± 6.9		0.29 ± 0.15	
Koyanagi, 2018 (*p*) ~															
Optovue	3 × 3	RP	73 (0)	43 (13–68)	0.161	0 (−0.2–0.7)	NA	Foveal: 27.1 (11.9–45.8) Parafov: 43.8 (34.6–54.6)	Foveal: 0.049 Parafov: <0.001	Foveal 24.5 (8.32–45.8) Parafov: 50.1 (39.7–61.1)	Foveal: 0.757 Parafov: <0.001	NA		Sup: 0.23 (0.08–1.05) Deep: 0.24 (0.09–1.10)	Sup: 0.3 Deep: 0.9
		Control	36	38 (27–61)		N/A		Foveal: 29.1 (22.2–40.1) Parafov: 54.7 (41.0–61.1)		Foveal 24.7 (17.7–34.1) Parafov: 61.7 (55.0–65.5)				Sup: 0.23 (0.09–0.37) Deep: 0.25 (0.11–0.45)	
Inooka, 2018 (R)															
Cirrus	3 × 3	RP	53 ^	48.3 ± 17.3	0.32	0.2 ± 0.26	<0.001	PD: 0.3854 ± 0.0166 VLD: 20.2 ± 1.2	PD: <0.001 VLD: <0.001	PD: 0.293 ± 0.048 VLD: 14.8 ± 2.7	PD: <0.001 VLD: <0.001	NA		0.309 ± 0.091	<0.001
		Control	46	52.7 ± 15.4		−0.001 ± 0.02		PD: 0.4166 ± 0.0080 VLD: 22.6 ± 0.8		PD: 0.348 ± 0.030 VLD: 18.4 ± 1.8				0.231 ± 0.065	
Takagi, 2018 (*p*)															
Optovue	3 × 3	RP	50 (0)	46.8 ± 12.6	0.33	0.11 ± 0.07	0.69	Flow area: 3.99 ± 0.38	0.007	Flow area: 4.06 ± 0.71	0.004	Flow area: 5.43 ± 0.17	0.353	Sup: 0.30 ± 0.09 Deep: 0.41 ± 0.13	Sup: 0.006 Deep: 0.2
		Control	22	50.3 ± 10.0		0.08 ± 0.05		Flow area: 4.32 ± 0.27		Flow area: 4.44 ± 0.37		Flow area: 5.47 ± 0.13		Sup: 0.36 ± 0.07 Deep: 0.42 ± 0.09	
Wang, 2019 (*p*)															
Cirrus	3 × 3	RP	40	38.7 ± 10.5	NA	NA		VAD: Fovea: 20.5 ± 5.4 Temp: 35.5 ± 4.2 Sup: 36.9 ± 3.8 Nasal: 36.6 ± 3.8 Inf: 36.7 ± 4.2	All: <0.001	NA		NA		0.6 ± 0.4	<0.01
		Control	26	42.3 ± 15.7				VAD: Fovea: 27.5 ± 5.5 Temp: 45.1 ± 1.8 Sup: 46.6 ± 1.8 Nasal: 45.8 ± 1.8 Inf: 45.9 ± 1.9						0.3 ± 0.1	
Hagag, 2019 (*p*)															
Optovue (projection resolved)	6 × 6	RP	20 (0)	49.6 ± 22.8	NA	0.11 ± 0.16	<0.002	SVC perifovea: 65.9 ± 4.7	0.56	ICP perifovea: 43.7 ± 7.3 DCP perifovea: 17.7 ± 7.7	ICP: 0.1 DCP: <0.001	NA		NA	
		Control	34	48.5 ± 23.7		−0.04 ± 0.09		SVC perifovea: 65.7 ± 5.2		ICP perifov: 46.7 ± 7.1 DCP perifovea: 25.9 ± 5.7					
Guduru, 2018 (*p*)															
Cirrus	6 × 6	RP	70	28.5 ± 13.2	0.8	0.32	NA	NA		NA		FV area: 0.33 ± 0.12	<0.001	NA	
		Control	37	29.1 ± 5.9		NA						FV area: 0.18 ± 0.1			

OCTA: optical coherence tomography angiography; CME: cystoid macular edema; BCVA: best corrected visual acuity; SCP: superficial capillary plexus; VD: vessel density; DCP: deep capillary plexus; CC: choriocapillaris; FAZ: foveal avascular zone; SD: standard deviation; *p*: prospective; R: retrospective; NA: not available; PD: perfusion density; VLD: vessel length density; VAD: vessel area density; FV: flow void ^+^ If available * Unless specified, values reported are for whole *en* face image ~ All values in this study expressed as median (range) ^ Under methods, eyes with segmentation errors caused by macular edema were excluded. Thus, it is not clear if all eyes with macular edema were excluded.

**Table 3:** Summary of microvascular changes reported in the literature comparing Stargardt patients and unaffected controls.

OCTA	Size of OCTA Images (mm)	Groups	N, Eyes	Age		BCVA		SCP VD *		DCP VD *		CC VD		FAZ Size	
				Mean Years ± SD	*p*-Value	Mean logMAR ± SD	*p*-Value	Mean % ± SD	*p*-Value	Mean % ± SD	*p*-Value	Mean % ± SD	*p*-Value	Mean mm^2^ ± SD	*p*-Value
Battaglia Parodi, 2017 (*p*)															
Topcon	3 × 3	STGD	36	33 ± 5.7	NA	0.6 ± 0.3	NA	30.2 ± 6.2	0.0002	30.3 ± 8.1	<0.001	50.9 ± 4.9	0.02	Sup: 0.43 ± 0.2 Deep: 0.67 ± 0.4	Sup: 0.012 Deep: 0.2
		Control	36	31.8 ± 7.6		0.0 ± 0.0		63.5 ± 4.2		39.9 ± 4.5		54.2 ± 5.7		Sup: 0.27 ± 0.13 Deep: 0.55 ± 0.2	
Mastropasqua, 2017 (*p*)															
Optovue	3 × 3	STGD	24	47.6 ± 15.6	1.0	0.9 ± 0.1	<0.001	Foveal: 27.25 ± 10.19 Parafoveal: 46.34 ± 4.04	Foveal: 0.153 Parafoveal: <0.001	Foveal: 37.52 ± 9.51 Parafoveal: 47.38 ± 4.25	Foveal: 0.015 Parafoveal: <0.001	Foveal: 54.87 ± 24.84 Parafoveal: 60.63 ± 6.46	Foveal: <0.001 Parafoveal: <0.001	NA	
		Control	24	45.3 ± 12.1		0.0 ± 0.5		Foveal: 31.44 ± 5.41 Parafoveal: 52.55 ± 2.94		Foveal: 29.68 ± 0.742 Parafoveal: 59.09 ± 2.79		Foveal: 27.51 ± 5.37 Parafoveal: 67.11 ± 1.40			

OCTA: optical coherence tomography angiography; CME: cystoid macular edema; BCVA: best corrected visual acuity; SCP: superficial capillary plexus; VD: vessel density; DCP: deep capillary plexus; CC: choriocapillaris; FAZ: foveal avascular zone; SD: standard deviation; *p*: prospective; NA: not available * Unless specified, values reported are for whole *en* face image.
